# Editorial: Biomechanics in blood cell functions and diseases

**DOI:** 10.3389/fbioe.2024.1397772

**Published:** 2024-04-08

**Authors:** Jiangguo Lin, Cheng Dong, Jianhua Wu

**Affiliations:** ^1^ Medical Research Institute, Guangdong Provincial People’s Hospital (Guangdong Academy of Medical Sciences), Southern Medical University, Guangzhou, China; ^2^ Department of Biomedical Engineering, The Hong Kong Polytechnic University, Kowloon, Hong Kong SAR, China; ^3^ Institute of Biomechanics, School of Bioscience and Bioengineering, South China University of Technology, Guangzhou, China

**Keywords:** blood cells, biomechanics, biomechanical microenvironment, extracellular matrix, mechano-sensitive receptors, microfluidic device

The intricate interplay between blood cells and their biomechanical microenvironment is crucial for the proper functioning of physiological and pathophysiological processes. Mechanical cues wield significant influence over a spectrum of blood cell responses, ranging from activation and cytokine production to metabolism, proliferation, morphological alterations, and migration (Xu et al., 2021; Sun et al., 2022; Rogers et al., 2024). In recent years, the development of advanced high-throughput methods and devices has facilitated the precise quantification of the mechanical properties of blood cells, holding promising potential for clinical diagnosis and drug screening. Concurrently, endeavors have been dedicated to targeting the extracellular matrix (Jiang et al., 2022) and engineering mechano-sensitive receptors (Zhao et al., 2022) to enhance therapeutic outcomes.

This Research Topic aims to shed new light on the mechanobiology of blood cells in both physiological and pathological conditions, featuring four peer-reviewed articles, including one original research article and three comprehensive reviews.

The deformability of blood cells is intricately linked to various physiological and pathophysiological behaviors. For instance, the high deformability of Red Blood Cells (RBCs) enables them to navigate through narrow capillaries in the microcirculation. Neutrophils undergo significant morphological changes during transmigration through vessel walls to reach sites of infection or inflammation. Different subpopulations of blood cells exhibit distinct mechanical properties, sparking interest in understanding the correlation between these properties and their functions. Waugh et al. delved into this aspect by measuring the mechanical properties of naïve or activated T cells and CD8^+^ cells using micropipette aspiration. Their analysis covered cortical tension, cell volume, initial cell entry magnitude, characteristic viscosity, and shear thinning coefficient. Activation was found to increase cellular resistance to deformation, broadening the distribution of cell properties. Reduced deformability in activated T cells could impede efficient trafficking to target tissue sites, highlighting the potential clinical implications of these findings. Other techniques such as Atomic Force Microscopy and laser tweezers have also been employed, albeit with limited throughput. The microfluidic-based approach emerges as a promising high-throughput tool for clinical measurement of blood cell deformability. An et al. reviewed the recent advancements in microfluidics for measuring blood cell deformation. The authors first introduced the principles of microfluidic-based cell deformation measurement, including different types and approaches of microfluidic. Next, they focused on the applications of microfluidic approaches in clinical diagnosis, such as the deformation studies on RBCs, stem cells, and cancer cells. Although microfluidic-based approaches are primarily used in laboratory setting, their translational impact was discussed.

The mechano-microenvironment significantly influences blood cell functions, and research over the past decades has scrutinized the mechanisms and mechano-transduction signaling pathways guiding blood cells’ responses to mechanical cues. In an effort to enhance outcomes, recent studies have targeted the mechano-microenvironment, including the extracellular matrix, and engineered mechano-sensitive receptors such as integrins. Zhao et al. explored immune-mechanobiology, focusing on how mechanosensitive receptors, particularly T cell receptors (TCR) and integrins, trigger T cell immune responses. They also discussed the mechanical immune-engineering strategies employed in T cell immunity.

In the next review, Ouyang et al. focused on the advances in foam cell formation in atherosclerosis and the regulation of atherosclerotic plaque and lipid metabolism by mechanical forces. Macrophages and smooth muscle cells are believed the main cell origins of foam cells, while evidence showed that foam cells are derived from endothelial cells as well. Atherosclerotic plaques are not random but distributed at the bend and bifurcation of the arterial tree, suggesting mechanical forces play an important role in atherosclerotic plaque formation. The authors emphasized the regulatory role of mechanical forces in lipid uptake by cells in the plaque.

In summary, this Research Topic provides novel insights into the mechanobiology of blood cells and how mechanical cues regulate the functions of blood cells. The work presented here points towards mechanical properties of blood cells, ECM, and mechano-sensitive receptors and signaling pathways emerging as attractive therapeutic targets. However, further interdisciplinary research is needed to successfully translate mechanobiology knowledge of blood cells into clinical practice.
